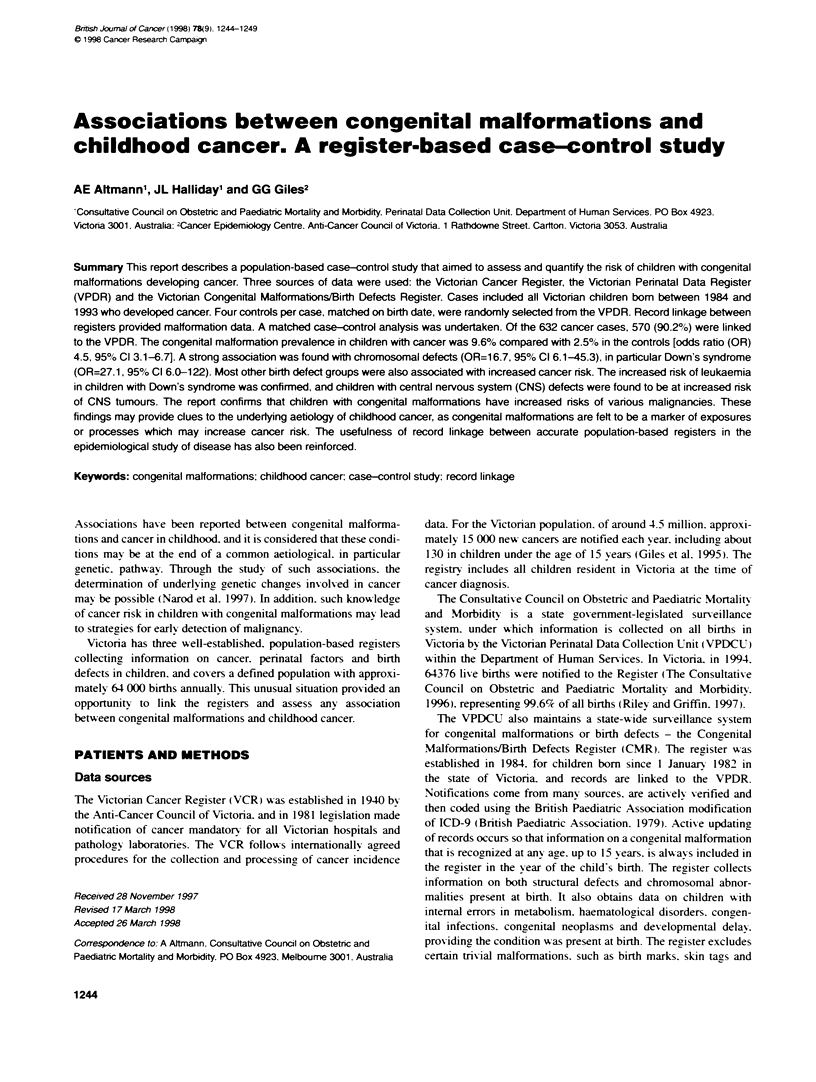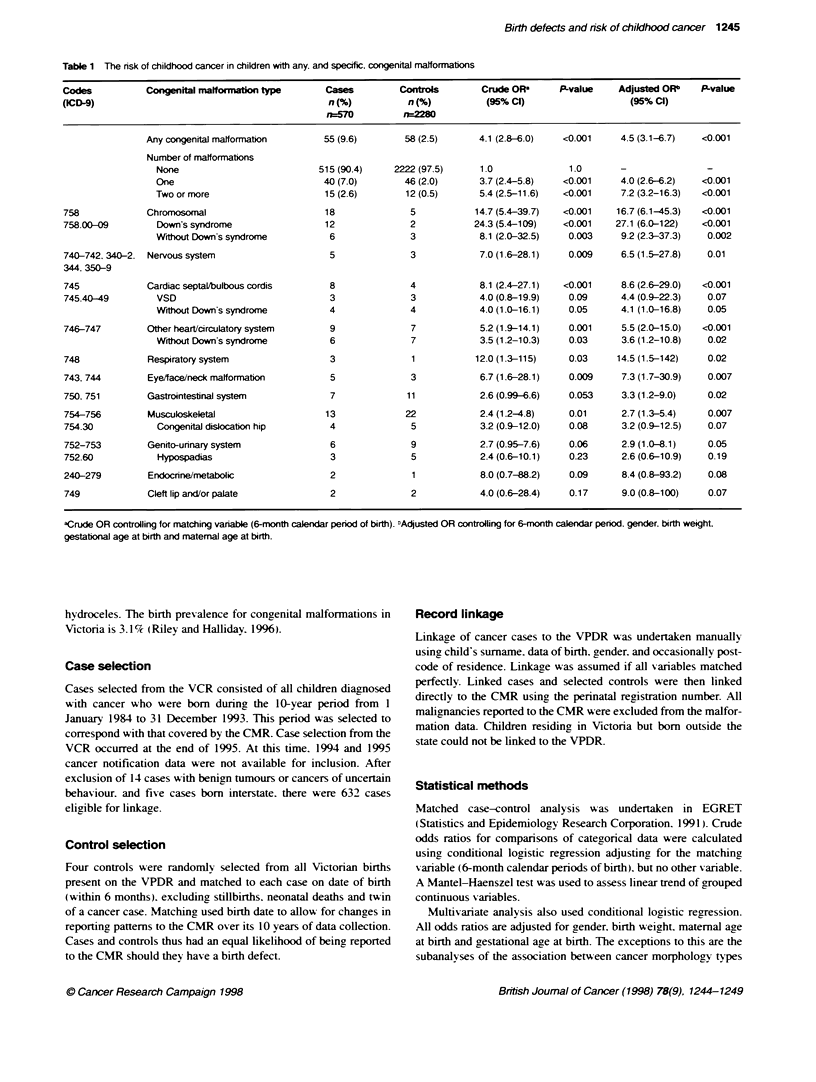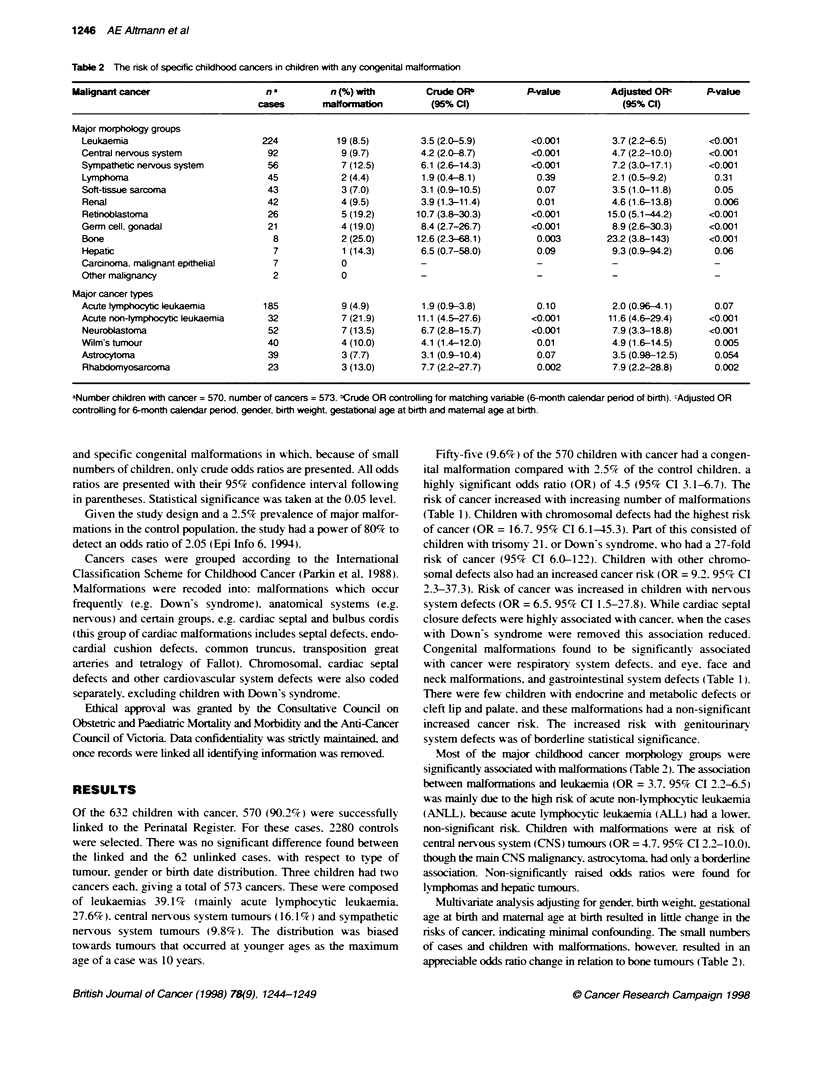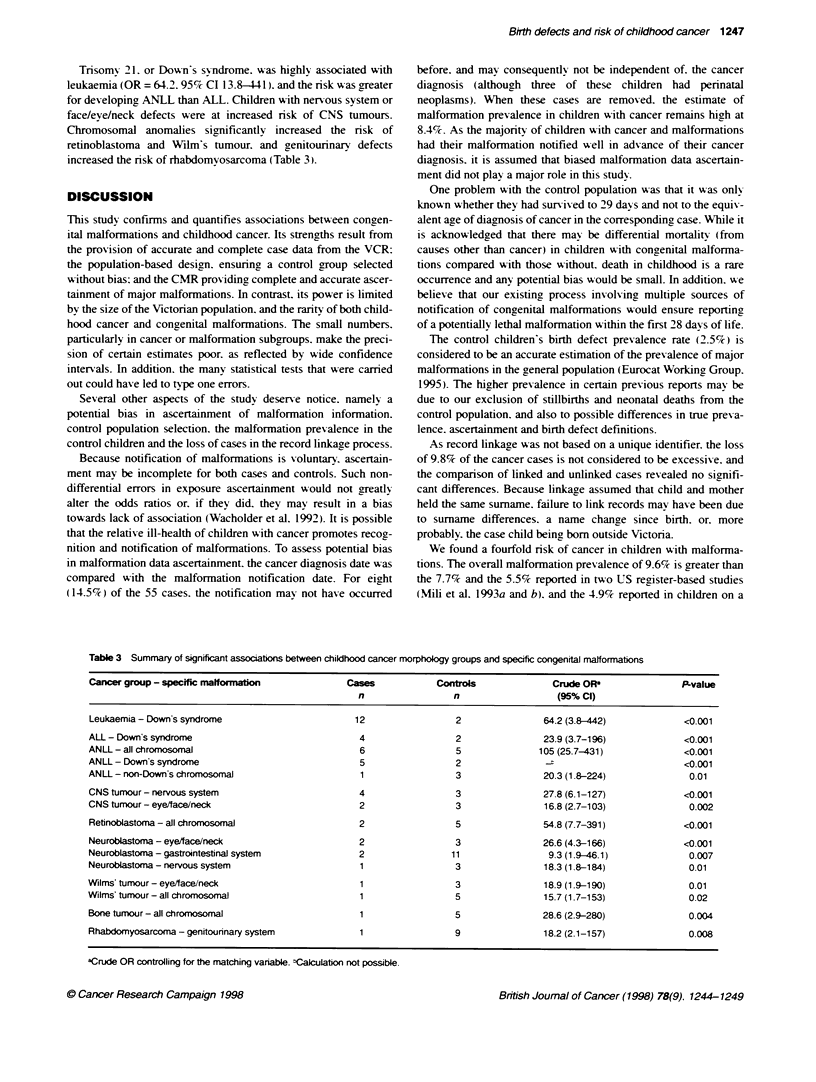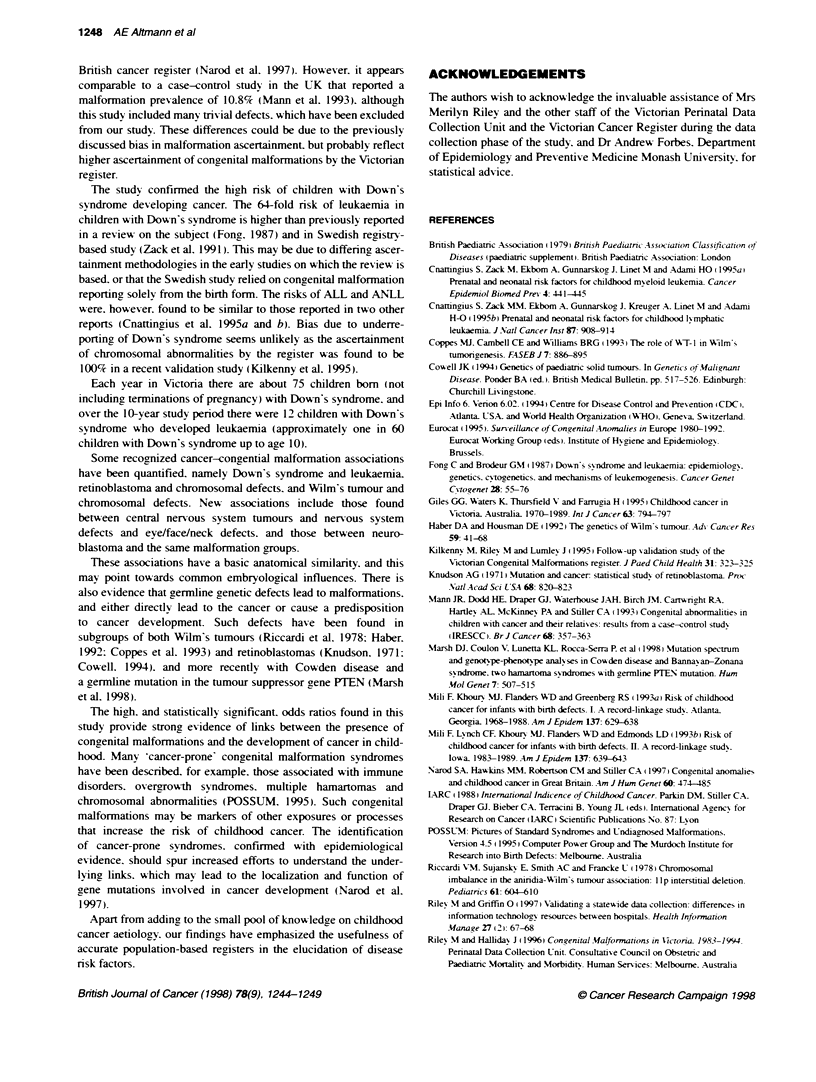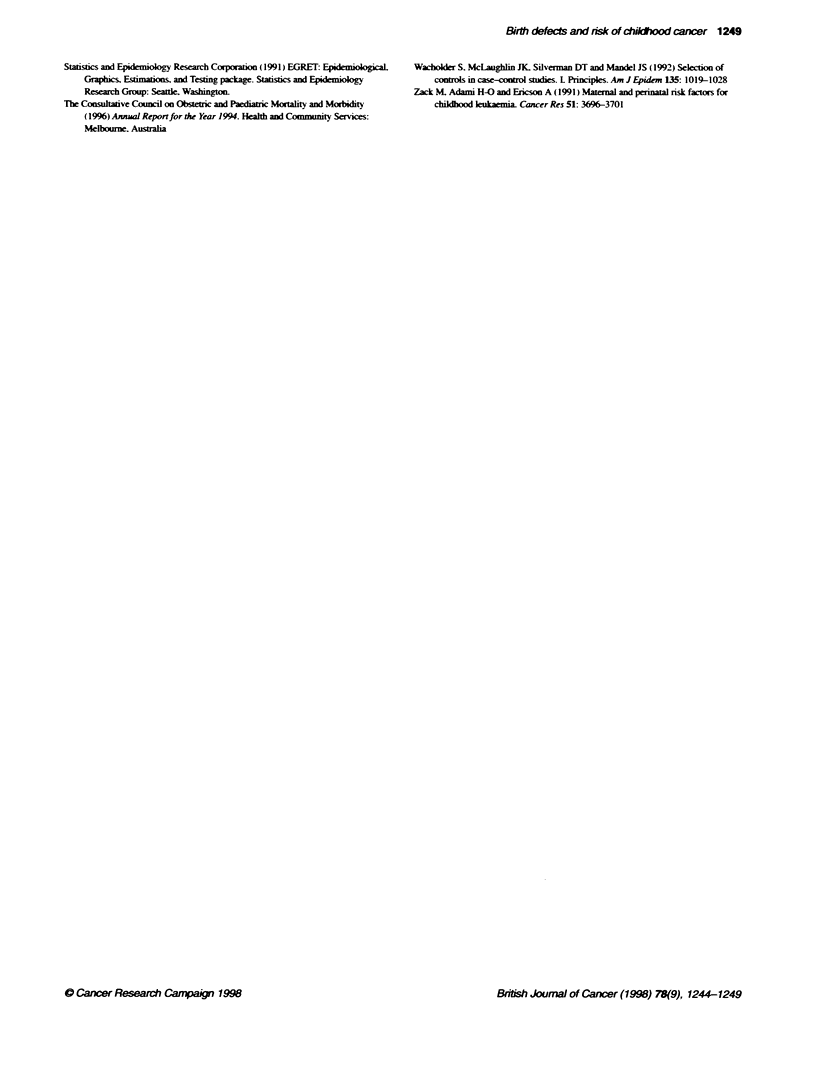# Associations between congenital malformations and childhood cancer. A register-based case-control study.

**DOI:** 10.1038/bjc.1998.662

**Published:** 1998-11

**Authors:** A. E. Altmann, J. L. Halliday, G. G. Giles

**Affiliations:** Consultative Council on Obstetric and Paediatric Mortality and Morbidity, Perinatal Data Collection Unit, Department of Human Services, Victoria, Australia.

## Abstract

This report describes a population-based case-control study that aimed to assess and quantify the risk of children with congenital malformations developing cancer. Three sources of data were used: the Victorian Cancer Register, the Victorian Perinatal Data Register (VPDR) and the Victorian Congenital Malformations/Birth Defects Register. Cases included all Victorian children born between 1984 and 1993 who developed cancer. Four controls per case, matched on birth date, were randomly selected from the VPDR. Record linkage between registers provided malformation data. A matched case-control analysis was undertaken. Of the 632 cancer cases, 570 (90.2%) were linked to the VPDR. The congenital malformation prevalence in children with cancer was 9.6% compared with 2.5% in the controls [odds ratio (OR) 4.5, 95% CI 3.1-6.7]. A strong association was found with chromosomal defects (OR=16.7, 95% CI 6.1-45.3), in particular Down's syndrome (OR=27.1, 95% CI 6.0-122). Most other birth defect groups were also associated with increased cancer risk. The increased risk of leukaemia in children with Down's syndrome was confirmed, and children with central nervous system (CNS) defects were found to be at increased risk of CNS tumours. The report confirms that children with congenital malformations have increased risks of various malignancies. These findings may provide clues to the underlying aetiology of childhood cancer, as congenital malformations are felt to be a marker of exposures or processes which may increase cancer risk. The usefulness of record linkage between accurate population-based registers in the epidemiological study of disease has also been reinforced.


British Journal of Cancer (1998) 78(9). 1244–1249
© 1998 Cancer Research Campaign

### Associations between congenital malformations and childhood cancer. A register-based case–control study

**AE Altmann[1], JL Halliday[1] and GG Giles[2]**

[1]Consultative Council on Obstetric and Paediatric Mortality and Morbidity. Perinatal Data Collection Unit. Department of Human Services. PO Box 4923. Victoria 3001. Australia: [2]Cancer Epidemiology Centre. Anti-Cancer Council of Victoria. 1 Rathdowne Street. Carlton. Victoria 3053. Australia

**Summary** This report describes a population-based case–control study that aimed to assess and quantify the risk of children with congenital malformations developing cancer. Three sources of data were used: the Victorian Cancer Register, the Victorian Perinatal Data Register (VPDR) and the Victorian Congenital Malformations/Birth Defects Register. Cases included all Victorian children born between 1984 and 1993 who developed cancer. Four controls per case, matched on birth date, were randomly selected from the VPDR. Record linkage between registers provided malformation data. A matched case–control analysis was undertaken. Of the 632 cancer cases, 570 (90.2%) were linked to the VPDR. The congenital malformation prevalence in children with cancer was 9.6% compared with 2.5% in the controls [odds ratio (OR) 4.5, 95% CI 3.1–6.7]. A strong association was found with chromosomal defects (OR=16.7, 95% CI 6.1–45.3), in particular Down's syndrome (OR=27.1, 95% CI 6.0–122). Most other birth defect groups were also associated with increased cancer risk. The increased risk of leukaemia in children with Down's syndrome was confirmed, and children with central nervous system (CNS) defects were found to be at increased risk of CNS tumours. The report confirms that children with congenital malformations have increased risks of various malignancies. These findings may provide clues to the underlying aetiology of childhood cancer, as congenital malformations are felt to be a marker of exposures or processes which may increase cancer risk. The usefulness of record linkage between accurate population-based registers in the epidemiological study of disease has also been reinforced.

**Keywords:** congenital malformations; childhood cancer; case–control study; record linkage

Associations have been reported between congenital malformations and cancer in childhood. and it is considered that these conditions may be at the end of a common aetiological. in particular genetic. pathway. Through the study of such associations. the determination of underlying genetic changes involved in cancer may be possible (Narod et al. 1997). In addition. such knowledge of cancer risk in children with congenital malformations may lead to strategies for early detection of malignancy.

Victoria has three well-established. population-based registers collecting information on cancer. perinatal factors and birth defects in children. and covers a defined population with approximately 64 000 births annually. This unusual situation provided an opportunity to link the registers and assess any association between congenital malformations and childhood cancer.

### PATIENTS AND METHODS

### Data sources

The Victorian Cancer Register (VCR) was established in 1940 by the Anti-Cancer Council of Victoria. and in 1981 legislation made notification of cancer mandatory for all Victorian hospitals and pathology laboratories. The VCR follows internationally agreed procedures for the collection and processing of cancer incidence data. For the Victorian population. of around 4.5 million. approximately 15 000 new cancers are notified each year. including about 130 in children under the age of 15 years (Giles et al. 1995). The registry includes all children resident in Victoria at the time of cancer diagnosis.

The Consultative Council on Obstetric and Paediatric Mortality and Morbidity is a state government-legislated surveillance system. under which information is collected on all births in Victoria by the Victorian Perinatal Data Collection Unit (VPDCU) within the Department of Human Services. In Victoria. in 1994. 64376 live births were notified to the Register (The Consultative Council on Obstetric and Paediatric Mortality and Morbidity. 1996). representing 99.6% of all births (Riley and Griffin. 1997).

The VPDCU also maintains a state-wide surveillance system for congenital malformations or birth defects – the Congenital Malformations/Birth Defects Register (CMR). The register was established in 1984. for children born since 1 January 1982 in the state of Victoria. and records are linked to the VPDR. Notifications come from many sources. are actively verified and then coded using the British Paediatric Association modification of ICD-9 (British Paediatric Association. 1979). Active updating of records occurs so that information on a congenital malformation that is recognized at any age. up to 15 years. is always included in the register in the year of the child's birth. The register collects information on both structural defects and chromosomal abnormalities present at birth. It also obtains data on children with internal errors in metabolism. haematological disorders. congenital infections. congenital neoplasms and developmental delay. providing the condition was present at birth. The register excludes certain trivial malformations. such as birth marks. skin tags and

*Received 28 November 1997*
*Revised 17 March 1998*
*Accepted 26 March 1998*

*Correspondence to:* A Altmann. Consultative Council on Obstetric and Paediatric Mortality and Morbidity. PO Box 4923. Melbourne 3001. Australia

Table 1 The risk of childhood cancer in children with any, and specific, congenital malformations

| Codes (ICD-9) | Congenital malformation type | Cases n (%) n=570 | Controls n (%) n=2280 | Crude OR[a] (95% CI) | P-value | Adjusted OR[b] (95% CI) | P-value |
|---|---|---|---|---|---|---|---|
| | Any congenital malformation | 55 (9.6) | 58 (2.5) | 4.1 (2.8–6.0) | <0.001 | 4.5 (3.1–6.7) | <0.001 |
| | Number of malformations | | | | | | |
| | None | 515 (90.4) | 2222 (97.5) | 1.0 | 1.0 | – | – |
| | One | 40 (7.0) | 46 (2.0) | 3.7 (2.4–5.8) | <0.001 | 4.0 (2.6–6.2) | <0.001 |
| | Two or more | 15 (2.6) | 12 (0.5) | 5.4 (2.5–11.6) | <0.001 | 7.2 (3.2–16.3) | <0.001 |
| 758 | Chromosomal | 18 | 5 | 14.7 (5.4–39.7) | <0.001 | 16.7 (6.1–45.3) | <0.001 |
| 758.00–09 | Down's syndrome | 12 | 2 | 24.3 (5.4–109) | <0.001 | 27.1 (6.0–122) | <0.001 |
| | Without Down's syndrome | 6 | 3 | 8.1 (2.0–32.5) | 0.003 | 9.2 (2.3–37.3) | 0.002 |
| 740–742, 340–2, 344, 350–9 | Nervous system | 5 | 3 | 7.0 (1.6–28.1) | 0.009 | 6.5 (1.5–27.8) | 0.01 |
| 745 | Cardiac septal/bulbous cordis | 8 | 4 | 8.1 (2.4–27.1) | <0.001 | 8.6 (2.6–29.0) | <0.001 |
| 745.40–49 | VSD | 3 | 3 | 4.0 (0.8–19.9) | 0.09 | 4.4 (0.9–22.3) | 0.07 |
| | Without Down's syndrome | 4 | 4 | 4.0 (1.0–16.1) | 0.05 | 4.1 (1.0–16.8) | 0.05 |
| 746–747 | Other heart/circulatory system | 9 | 7 | 5.2 (1.9–14.1) | 0.001 | 5.5 (2.0–15.0) | <0.001 |
| | Without Down's syndrome | 6 | 7 | 3.5 (1.2–10.3) | 0.03 | 3.6 (1.2–10.8) | 0.02 |
| 748 | Respiratory system | 3 | 1 | 12.0 (1.3–115) | 0.03 | 14.5 (1.5–142) | 0.02 |
| 743, 744 | Eye/face/neck malformation | 5 | 3 | 6.7 (1.6–28.1) | 0.009 | 7.3 (1.7–30.9) | 0.007 |
| 750, 751 | Gastrointestinal system | 7 | 11 | 2.6 (0.99–6.6) | 0.053 | 3.3 (1.2–9.0) | 0.02 |
| 754–756 | Musculoskeletal | 13 | 22 | 2.4 (1.2–4.8) | 0.01 | 2.7 (1.3–5.4) | 0.007 |
| 754.30 | Congenital dislocation hip | 4 | 5 | 3.2 (0.9–12.0) | 0.08 | 3.2 (0.9–12.5) | 0.07 |
| 752–753 | Genito-urinary system | 6 | 9 | 2.7 (0.95–7.6) | 0.06 | 2.9 (1.0–8.1) | 0.05 |
| 752.60 | Hypospadias | 3 | 5 | 2.4 (0.6–10.1) | 0.23 | 2.6 (0.6–10.9) | 0.19 |
| 240–279 | Endocrine/metabolic | 2 | 1 | 8.0 (0.7–88.2) | 0.09 | 8.4 (0.8–93.2) | 0.08 |
| 749 | Cleft lip and/or palate | 2 | 2 | 4.0 (0.6–28.4) | 0.17 | 9.0 (0.8–100) | 0.07 |

[a]Crude OR controlling for matching variable (6-month calendar period of birth). [b]Adjusted OR controlling for 6-month calendar period, gender, birth weight, gestational age at birth and maternal age at birth.

hydroceles. The birth prevalence for congenital malformations in Victoria is 3.1% (Riley and Halliday, 1996).

### Case selection

Cases selected from the VCR consisted of all children diagnosed with cancer who were born during the 10-year period from 1 January 1984 to 31 December 1993. This period was selected to correspond with that covered by the CMR. Case selection from the VCR occurred at the end of 1995. At this time, 1994 and 1995 cancer notification data were not available for inclusion. After exclusion of 14 cases with benign tumours or cancers of uncertain behaviour, and five cases born interstate, there were 632 cases eligible for linkage.

### Control selection

Four controls were randomly selected from all Victorian births present on the VPDR and matched to each case on date of birth (within 6 months), excluding stillbirths, neonatal deaths and twin of a cancer case. Matching used birth date to allow for changes in reporting patterns to the CMR over its 10 years of data collection. Cases and controls thus had an equal likelihood of being reported to the CMR should they have a birth defect.

### Record linkage

Linkage of cancer cases to the VPDR was undertaken manually using child's surname, data of birth, gender, and occasionally postcode of residence. Linkage was assumed if all variables matched perfectly. Linked cases and selected controls were then linked directly to the CMR using the perinatal registration number. All malignancies reported to the CMR were excluded from the malformation data. Children residing in Victoria but born outside the state could not be linked to the VPDR.

### Statistical methods

Matched case–control analysis was undertaken in EGRET (Statistics and Epidemiology Research Corporation, 1991). Crude odds ratios for comparisons of categorical data were calculated using conditional logistic regression adjusting for the matching variable (6-month calendar periods of birth), but no other variable. A Mantel–Haenszel test was used to assess linear trend of grouped continuous variables.

Multivariate analysis also used conditional logistic regression. All odds ratios are adjusted for gender, birth weight, maternal age at birth and gestational age at birth. The exceptions to this are the subanalyses of the association between cancer morphology types

© Cancer Research Campaign 1998

**Table 2** The risk of specific childhood cancers in children with any congenital malformation

| Malignant cancer | $n$[a] cases | $n$ (%) with malformation | Crude OR[b] (95% CI) | P-value | Adjusted OR[c] (95% CI) | P-value |
|---|---|---|---|---|---|---|
| Major morphology groups | | | | | | |
| Leukaemia | 224 | 19 (8.5) | 3.5 (2.0–5.9) | <0.001 | 3.7 (2.2–6.5) | <0.001 |
| Central nervous system | 92 | 9 (9.7) | 4.2 (2.0–8.7) | <0.001 | 4.7 (2.2–10.0) | <0.001 |
| Sympathetic nervous system | 56 | 7 (12.5) | 6.1 (2.6–14.3) | <0.001 | 7.2 (3.0–17.1) | <0.001 |
| Lymphoma | 45 | 2 (4.4) | 1.9 (0.4–8.1) | 0.39 | 2.1 (0.5–9.2) | 0.31 |
| Soft-tissue sarcoma | 43 | 3 (7.0) | 3.1 (0.9–10.5) | 0.07 | 3.5 (1.0–11.8) | 0.05 |
| Renal | 42 | 4 (9.5) | 3.9 (1.3–11.4) | 0.01 | 4.6 (1.6–13.8) | 0.006 |
| Retinoblastoma | 26 | 5 (19.2) | 10.7 (3.8–30.3) | <0.001 | 15.0 (5.1–44.2) | <0.001 |
| Germ cell, gonadal | 21 | 4 (19.0) | 8.4 (2.7–26.7) | <0.001 | 8.9 (2.6–30.3) | <0.001 |
| Bone | 8 | 2 (25.0) | 12.6 (2.3–68.1) | 0.003 | 23.2 (3.8–143) | <0.001 |
| Hepatic | 7 | 1 (14.3) | 6.5 (0.7–58.0) | 0.09 | 9.3 (0.9–94.2) | 0.06 |
| Carcinoma, malignant epithelial | 7 | 0 | – | – | – | – |
| Other malignancy | 2 | 0 | – | – | – | – |
| Major cancer types | | | | | | |
| Acute lymphocytic leukaemia | 185 | 9 (4.9) | 1.9 (0.9–3.8) | 0.10 | 2.0 (0.96–4.1) | 0.07 |
| Acute non-lymphocytic leukaemia | 32 | 7 (21.9) | 11.1 (4.5–27.6) | <0.001 | 11.6 (4.6–29.4) | <0.001 |
| Neuroblastoma | 52 | 7 (13.5) | 6.7 (2.8–15.7) | <0.001 | 7.9 (3.3–18.8) | <0.001 |
| Wilm's tumour | 40 | 4 (10.0) | 4.1 (1.4–12.0) | 0.01 | 4.9 (1.6–14.5) | 0.005 |
| Astrocytoma | 39 | 3 (7.7) | 3.1 (0.9–10.4) | 0.07 | 3.5 (0.98–12.5) | 0.054 |
| Rhabdomyosarcoma | 23 | 3 (13.0) | 7.7 (2.2–27.7) | 0.002 | 7.9 (2.2–28.8) | 0.002 |

[a]Number children with cancer = 570, number of cancers = 573. [b]Crude OR controlling for matching variable (6-month calendar period of birth). [c]Adjusted OR controlling for 6-month calendar period, gender, birth weight, gestational age at birth and maternal age at birth.

and specific congenital malformations in which, because of small numbers of children, only crude odds ratios are presented. All odds ratios are presented with their 95% confidence interval following in parentheses. Statistical significance was taken at the 0.05 level.

Given the study design and a 2.5% prevalence of major malformations in the control population, the study had a power of 80% to detect an odds ratio of 2.05 (Epi Info 6, 1994).

Cancers cases were grouped according to the International Classification Scheme for Childhood Cancer (Parkin et al, 1988). Malformations were recoded into: malformations which occur frequently (e.g. Down's syndrome), anatomical systems (e.g. nervous) and certain groups, e.g. cardiac septal and bulbus cordis (this group of cardiac malformations includes septal defects, endocardial cushion defects, common truncus, transposition great arteries and tetralogy of Fallot). Chromosomal, cardiac septal defects and other cardiovascular system defects were also coded separately, excluding children with Down's syndrome.

Ethical approval was granted by the Consultative Council on Obstetric and Paediatric Mortality and Morbidity and the Anti-Cancer Council of Victoria. Data confidentiality was strictly maintained, and once records were linked all identifying information was removed.

### RESULTS

Of the 632 children with cancer, 570 (90.2%) were successfully linked to the Perinatal Register. For these cases, 2280 controls were selected. There was no significant difference found between the linked and the 62 unlinked cases, with respect to type of tumour, gender or birth date distribution. Three children had two cancers each, giving a total of 573 cancers. These were composed of leukaemias 39.1% (mainly acute lymphocytic leukaemia, 27.6%), central nervous system tumours (16.1%) and sympathetic nervous system tumours (9.8%). The distribution was biased towards tumours that occurred at younger ages as the maximum age of a case was 10 years.

Fifty-five (9.6%) of the 570 children with cancer had a congenital malformation compared with 2.5% of the control children, a highly significant odds ratio (OR) of 4.5 (95% CI 3.1–6.7). The risk of cancer increased with increasing number of malformations (Table 1). Children with chromosomal defects had the highest risk of cancer (OR = 16.7, 95% CI 6.1–45.3). Part of this consisted of children with trisomy 21, or Down's syndrome, who had a 27-fold risk of cancer (95% CI 6.0–122). Children with other chromosomal defects also had an increased cancer risk (OR = 9.2, 95% CI 2.3–37.3). Risk of cancer was increased in children with nervous system defects (OR = 6.5, 95% CI 1.5–27.8). While cardiac septal closure defects were highly associated with cancer, when the cases with Down's syndrome were removed this association reduced. Congenital malformations found to be significantly associated with cancer were respiratory system defects, and eye, face and neck malformations, and gastrointestinal system defects (Table 1). There were few children with endocrine and metabolic defects or cleft lip and palate, and these malformations had a non-significant increased cancer risk. The increased risk with genitourinary system defects was of borderline statistical significance.

Most of the major childhood cancer morphology groups were significantly associated with malformations (Table 2). The association between malformations and leukaemia (OR = 3.7, 95% CI 2.2–6.5) was mainly due to the high risk of acute non-lymphocytic leukaemia (ANLL), because acute lymphocytic leukaemia (ALL) had a lower, non-significant risk. Children with malformations were at risk of central nervous system (CNS) tumours (OR = 4.7, 95% CI 2.2–10.0), though the main CNS malignancy, astrocytoma, had only a borderline association. Non-significantly raised odds ratios were found for lymphomas and hepatic tumours.

Multivariate analysis adjusting for gender, birth weight, gestational age at birth and maternal age at birth resulted in little change in the risks of cancer, indicating minimal confounding. The small numbers of cases and children with malformations, however, resulted in an appreciable odds ratio change in relation to bone tumours (Table 2).

© Cancer Research Campaign 1998

Trisomy 21, or Down's syndrome, was highly associated with leukaemia (OR = 64.2, 95% CI 13.8–441), and the risk was greater for developing ANLL than ALL. Children with nervous system or face/eye/neck defects were at increased risk of CNS tumours. Chromosomal anomalies significantly increased the risk of retinoblastoma and Wilm's tumour, and genitourinary defects increased the risk of rhabdomyosarcoma (Table 3).

### DISCUSSION

This study confirms and quantifies associations between congenital malformations and childhood cancer. Its strengths result from the provision of accurate and complete case data from the VCR; the population-based design, ensuring a control group selected without bias; and the CMR providing complete and accurate ascertainment of major malformations. In contrast, its power is limited by the size of the Victorian population, and the rarity of both childhood cancer and congenital malformations. The small numbers, particularly in cancer or malformation subgroups, make the precision of certain estimates poor, as reflected by wide confidence intervals. In addition, the many statistical tests that were carried out could have led to type one errors.

Several other aspects of the study deserve notice, namely a potential bias in ascertainment of malformation information, control population selection, the malformation prevalence in the control children and the loss of cases in the record linkage process.

Because notification of malformations is voluntary, ascertainment may be incomplete for both cases and controls. Such non-differential errors in exposure ascertainment would not greatly alter the odds ratios or, if they did, they may result in a bias towards lack of association (Wacholder et al, 1992). It is possible that the relative ill-health of children with cancer promotes recognition and notification of malformations. To assess potential bias in malformation data ascertainment, the cancer diagnosis date was compared with the malformation notification date. For eight (14.5%) of the 55 cases, the notification may not have occurred before, and may consequently not be independent of, the cancer diagnosis (although three of these children had perinatal neoplasms). When these cases are removed, the estimate of malformation prevalence in children with cancer remains high at 8.4%. As the majority of children with cancer and malformations had their malformation notified well in advance of their cancer diagnosis, it is assumed that biased malformation data ascertainment did not play a major role in this study.

One problem with the control population was that it was only known whether they had survived to 29 days and not to the equivalent age of diagnosis of cancer in the corresponding case. While it is acknowledged that there may be differential mortality (from causes other than cancer) in children with congenital malformations compared with those without, death in childhood is a rare occurrence and any potential bias would be small. In addition, we believe that our existing process involving multiple sources of notification of congenital malformations would ensure reporting of a potentially lethal malformation within the first 28 days of life.

The control children's birth defect prevalence rate (2.5%) is considered to be an accurate estimation of the prevalence of major malformations in the general population (Eurocat Working Group, 1995). The higher prevalence in certain previous reports may be due to our exclusion of stillbirths and neonatal deaths from the control population, and also to possible differences in true prevalence, ascertainment and birth defect definitions.

As record linkage was not based on a unique identifier, the loss of 9.8% of the cancer cases is not considered to be excessive, and the comparison of linked and unlinked cases revealed no significant differences. Because linkage assumed that child and mother held the same surname, failure to link records may have been due to surname differences, a name change since birth, or, more probably, the case child being born outside Victoria.

We found a fourfold risk of cancer in children with malformations. The overall malformation prevalence of 9.6% is greater than the 7.7% and the 5.5% reported in two US register-based studies (Mili et al, 1993*a* and *b*), and the 4.9% reported in children on a

**Table 3** Summary of significant associations between childhood cancer morphology groups and specific congenital malformations

| Cancer group – specific malformation | Cases *n* | Controls *n* | Crude OR[a] (95% CI) | *P*-value |
|---|---|---|---|---|
| Leukaemia – Down's syndrome | 12 | 2 | 64.2 (3.8–442) | <0.001 |
| ALL – Down's syndrome | 4 | 2 | 23.9 (3.7–196) | <0.001 |
| ANLL – all chromosomal | 6 | 5 | 105 (25.7–431) | <0.001 |
| ANLL – Down's syndrome | 5 | 2 | –[b] | <0.001 |
| ANLL – non-Down's chromosomal | 1 | 3 | 20.3 (1.8–224) | 0.01 |
| CNS tumour – nervous system | 4 | 3 | 27.8 (6.1–127) | <0.001 |
| CNS tumour – eye/face/neck | 2 | 3 | 16.8 (2.7–103) | 0.002 |
| Retinoblastoma – all chromosomal | 2 | 5 | 54.8 (7.7–391) | <0.001 |
| Neuroblastoma – eye/face/neck | 2 | 3 | 26.6 (4.3–166) | <0.001 |
| Neuroblastoma – gastrointestinal system | 2 | 11 | 9.3 (1.9–46.1) | 0.007 |
| Neuroblastoma – nervous system | 1 | 3 | 18.3 (1.8–184) | 0.01 |
| Wilms' tumour – eye/face/neck | 1 | 3 | 18.9 (1.9–190) | 0.01 |
| Wilms' tumour – all chromosomal | 1 | 5 | 15.7 (1.7–153) | 0.02 |
| Bone tumour – all chromosomal | 1 | 5 | 28.6 (2.9–280) | 0.004 |
| Rhabdomyosarcoma – genitourinary system | 1 | 9 | 18.2 (2.1–157) | 0.008 |

[a]Crude OR controlling for the matching variable. [b]Calculation not possible.

© Cancer Research Campaign 1998

British cancer register (Narod et al. 1997). However, it appears comparable to a case–control study in the UK that reported a malformation prevalence of 10.8% (Mann et al. 1993), although this study included many trivial defects, which have been excluded from our study. These differences could be due to the previously discussed bias in malformation ascertainment, but probably reflect higher ascertainment of congenital malformations by the Victorian register.

The study confirmed the high risk of children with Down's syndrome developing cancer. The 64-fold risk of leukaemia in children with Down's syndrome is higher than previously reported in a review on the subject (Fong, 1987) and in Swedish registry-based study (Zack et al. 1991). This may be due to differing ascertainment methodologies in the early studies on which the review is based, or that the Swedish study relied on congenital malformation reporting solely from the birth form. The risks of ALL and ANLL were, however, found to be similar to those reported in two other reports (Cnattingius et al. 1995*a* and *b*). Bias due to underreporting of Down's syndrome seems unlikely as the ascertainment of chromosomal abnormalities by the register was found to be 100% in a recent validation study (Kilkenny et al. 1995).

Each year in Victoria there are about 75 children born (not including terminations of pregnancy) with Down's syndrome, and over the 10-year study period there were 12 children with Down's syndrome who developed leukaemia (approximately one in 60 children with Down's syndrome up to age 10).

Some recognized cancer–congential malformation associations have been quantified, namely Down's syndrome and leukaemia, retinoblastoma and chromosomal defects, and Wilm's tumour and chromosomal defects. New associations include those found between central nervous system tumours and nervous system defects and eye/face/neck defects, and those between neuroblastoma and the same malformation groups.

These associations have a basic anatomical similarity, and this may point towards common embryological influences. There is also evidence that germline genetic defects lead to malformations, and either directly lead to the cancer or cause a predisposition to cancer development. Such defects have been found in subgroups of both Wilm's tumours (Riccardi et al. 1978; Haber, 1992; Coppes et al. 1993) and retinoblastomas (Knudson, 1971; Cowell, 1994), and more recently with Cowden disease and a germline mutation in the tumour suppressor gene PTEN (Marsh et al. 1998).

The high, and statistically significant, odds ratios found in this study provide strong evidence of links between the presence of congenital malformations and the development of cancer in childhood. Many 'cancer-prone' congenital malformation syndromes have been described, for example, those associated with immune disorders, overgrowth syndromes, multiple hamartomas and chromosomal abnormalities (POSSUM, 1995). Such congenital malformations may be markers of other exposures or processes that increase the risk of childhood cancer. The identification of cancer-prone syndromes, confirmed with epidemiological evidence, should spur increased efforts to understand the underlying links, which may lead to the localization and function of gene mutations involved in cancer development (Narod et al. 1997).

Apart from adding to the small pool of knowledge on childhood cancer aetiology, our findings have emphasized the usefulness of accurate population-based registers in the elucidation of disease risk factors.

### ACKNOWLEDGEMENTS

The authors wish to acknowledge the invaluable assistance of Mrs Merilyn Riley and the other staff of the Victorian Perinatal Data Collection Unit and the Victorian Cancer Register during the data collection phase of the study, and Dr Andrew Forbes, Department of Epidemiology and Preventive Medicine Monash University, for statistical advice.

### REFERENCES

British Paediatric Association (1979) *British Paediatric Association Classification of Diseases* (paediatric supplement). British Paediatric Association: London

Cnattingius S, Zack M, Ekbom A, Gunnarskog J, Linet M and Adami HO (1995*a*) Prenatal and neonatal risk factors for childhood myeloid leukemia. *Cancer Epidemiol Biomed Prev* **4**: 441–445

Cnattingius S, Zack MM, Ekbom A, Gunnarskog J, Kreuger A, Linet M and Adami H-O (1995*b*) Prenatal and neonatal risk factors for childhood lymphatic leukaemia. *J Natl Cancer Inst* **87**: 908–914

Coppes MJ, Cambell CE and Williams BRG (1993) The role of WT-1 in Wilm's tumorigenesis. *FASEB J* **7**: 886–895

Cowell JK (1994) Genetics of paediatric solid tumours. In *Genetics of Malignant Disease*. Ponder BA (ed.). British Medical Bulletin, pp. 517–526. Edinburgh: Churchill Livingstone.

Epi Info 6, Verion 6.02, (1994) Centre for Disease Control and Prevention (CDC), Atlanta, USA, and World Health Organization (WHO), Geneva, Switzerland.

Eurocat (1995), *Surveillance of Congenital Anomalies in* Europe 1980–1992. Eurocat Working Group (eds). Institute of Hygiene and Epidemiology, Brussels.

Fong C and Brodeur GM (1987) Down's syndrome and leukaemia: epidemiology, genetics, cytogenetics, and mechanisms of leukemogenesis. *Cancer Genet Cytogenet* **28**: 55–76

Giles GG, Waters K, Thursfield V and Farrugia H (1995) Childhood cancer in Victoria, Australia, 1970–1989. *Int J Cancer* **63**: 794–797

Haber DA and Housman DE (1992) The genetics of Wilm's tumour. *Adv Cancer Res* **59**: 41–68

Kilkenny M, Riley M and Lumley J (1995) Follow-up validation study of the Victorian Congenital Malformations register. *J Paed Child Health* **31**: 323–325

Knudson AG (1971) Mutation and cancer: statistical study of retinoblastoma. *Proc Natl Acad Sci USA* **68**: 820–823

Mann JR, Dodd HE, Draper GJ, Waterhouse JAH, Birch JM, Cartwright RA, Hartley AL, McKinney PA and Stiller CA (1993) Congenital abnormalities in children with cancer and their relatives: results from a case–control study (IRESCC). *Br J Cancer* **68**: 357–363

Marsh DJ, Coulon V, Lunetta KL, Rocca-Serra P, et al (1998) Mutation spectrum and genotype-phenotype analyses in Cowden disease and Bannayan–Zonana syndrome, two hamartoma syndromes with germline PTEN mutation. *Hum Mol Genet* **7**: 507–515

Mili F, Khoury MJ, Flanders WD and Greenberg RS (1993*a*) Risk of childhood cancer for infants with birth defects. I. A record-linkage study, Atlanta, Georgia, 1968–1988. *Am J Epidem* **137**: 629–638

Mili F, Lynch CF, Khoury MJ, Flanders WD and Edmonds LD (1993*b*) Risk of childhood cancer for infants with birth defects. II. A record-linkage study, Iowa, 1983–1989. *Am J Epidem* **137**: 639–643

Narod SA, Hawkins MM, Robertson CM and Stiller CA (1997) Congenital anomalies and childhood cancer in Great Britain. *Am J Hum Genet* **60**: 474–485

IARC (1988) *International Indicence of Childhood Cancer*. Parkin DM, Stiller CA, Draper GJ, Bieber CA, Terracini B, Young JL (eds). International Agency for Research on Cancer (IARC) Scientific Publications No. 87: Lyon

POSSUM: Pictures of Standard Syndromes and Undiagnosed Malformations, Version 4.5 (1995) Computer Power Group and The Murdoch Institute for Research into Birth Defects: Melbourne, Australia

Riccardi VM, Sujansky E, Smith AC and Francke U (1978) Chromosomal imbalance in the aniridia-Wilm's tumour association: 11p interstitial deletion. *Pediatrics* **61**: 604–610

Riley M and Griffin O (1997) Validating a statewide data collection: differences in information technology resources between hospitals. *Health Information Manage* **27** (2): 67–68

Riley M and Halliday J (1996) *Congenital Malformations in Victoria, 1983–1994*. Perinatal Data Collection Unit, Consultative Council on Obstetric and Paediatric Mortality and Morbidity, Human Services: Melbourne, Australia

© Cancer Research Campaign 1998

Statistics and Epidemiology Research Corporation (1991) EGRET: Epidemiological, Graphics, Estimations, and Testing package. Statistics and Epidemiology Research Group: Seattle, Washington.

The Consultative Council on Obstetric and Paediatric Mortality and Morbidity (1996) *Annual Report for the Year 1994*. Health and Community Services: Melbourne, Australia

Wacholder S, McLaughlin JK, Silverman DT and Mandel JS (1992) Selection of controls in case–control studies. I. Principles. *Am J Epidem* **135**: 1019–1028

Zack M, Adami H-O and Ericson A (1991) Maternal and perinatal risk factors for childhood leukaemia. *Cancer Res* **51**: 3696–3701

© Cancer Research Campaign 1998